# Book review

**Published:** 2013

**Figure:**
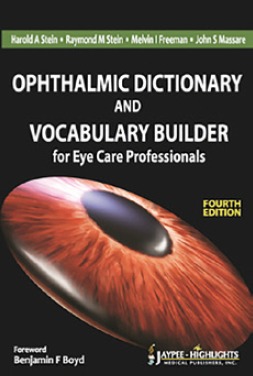
Ophthalmic Dictionary and Vocabulary Builder for Eye Care Professionals, 4th edition

Reviewed by **Nick Astbury**

This reference book informs the reader about the context and derivations of words, so helping to build up a rounded knowledge of the ophthalmic vocabulary. The book is illustrated throughout with line drawings and colour photographs.

Personally I miss seeing Snellen acuity expressed in metres (6/6) but I realise that this is an American text; however, it would be useful to include a LogMar equivalent under the estimation of visual acuity. A reference to small incision cataract surgery would also be helpful, considering its widespread use in low-and middle-income countries.

This remains a professionally written, practical and informative dictionary and vocabulary builder which would sit comfortably on the bookshelf of any ophthalmic technician, eye care professional, administrator, or editor.

**Cost:** UK £38. To order, visit **www.jpmedpub.com** and enter the discount code CEHJ23 to get a 20% discount, or email **orders@nbninternational.com** or call +44 1752 202-301 and give the same discount code. Valid until 31710/13.

## Subscriptions

Would you like to receive your own copy of the *Community Eye Health Journal?* Or have you changed address? Send your name, occupation, email address and home address to: Anita Shah, International Centre for Eye Health, London School of Hygiene and Tropical Medicine, London WC1E 7HT, UK. Email: **admin@cehjournal.org**

## Courses

**London School of Hygiene and Tropical Medicine, London, UK** MSc Public Health for Eye Care, starting September 2014. Read more about it and see what previous graduates say at **www.iceh.org.uk/display/WEB/MSc+Public+health+for+eye+care** To apply, **visit www.lshtm.ac.uk/study/masters/mscphec.html**

**German Jordanian University, Amman, Jordan**

Professional Diploma in Vision Rehabilitation (4 months, US $1,040) and MSc in Vision Rehabilitation (2 years, US $4,800). Open to optometrists, therapists, educators and rehabilitation workers. Courses start in September every year. Email: **vtc@gju.edu.jo** or visit **http://tinyurl.com/rehabcourse.**

**Community Eye Health Institute, University of Cape Town, South Africa**

Contact Zanele Magwa, Community Eye Health Institute, University of Cape Town, Private Bag 3, Rondebosch 7700, South Africa. Tel: +27 21 404 7735. Email: **ntombizanele.magwa@uct.ac.za**

**Kilimanjaro Centre for Community Ophthalmology (KCCO), Tanzania**

Contact Genes Mng'anya, KCCO Tanzania Limited PO Box 2254, Moshi, Tanzania. Tel: +255 27 275 3547 or visit **www.kcco.net**

**Lions SightFirst Eye Hospital, Nairobi, Kenya**

Small incision cataract surgery for ophthalmologists wishing to upgrade from ECCE. Duration: 6 weeks. Cost: US $1,000 for tuition and US $945 for accommodation and meals. Write to: The Training Coordinator, Lions Medical Training Centre, Lions SightFirst Eye Hospital, PO Box 66576-00800, Nairobi, Kenya. Tel:+254 20 418 32 39. Email: **training@lionsloresho.org**

## Online courses

### Aurosiksha

Free short online courses for eye care professionals to help them maintain skills and continue their professional development from Lions Aravind Institute of Community Ophthalmology (LAICO), India. Visit **www.aurosiksha.org**

### ORBIS CyberSight

Free courses on strabismus, cataract, paediatric ophthalmology, neuro-ophthalmology and the cornea are currently available. Registration is free. Visit **www.cybersight.org**

